# Case report: Efficacy of icotinib treatment in lung adenocarcinoma with esophageal squamous cell carcinoma: a rare case of double primary malignant tumors

**DOI:** 10.3389/fmed.2024.1266062

**Published:** 2024-03-28

**Authors:** Min Deng, Xiaoqing Li, Honghao Mu, Man Wei, Lan Sun

**Affiliations:** Department of Oncology, Bishan Hospital of Chongqing Medical University, Chongqing, China

**Keywords:** lung adenocarcinoma, esophageal squamous cell carcinoma, double primary malignant tumors, tyrosine kinase inhibitor, icotinib

## Abstract

**Background:**

Lung adenocarcinoma with esophageal squamous cell carcinoma is rare and the prognosis is poor, therefore there is an urgent need to improve this situation. The objective of this study was to explore the effect of first-generation tyrosine kinase inhibitors (TKIs) in the patient of the double primary malignant tumors.

**Case report:**

We report a case of lung adenocarcinoma with esophageal squamous cell carcinoma treated by icotininb after five-year follow-up. A 71-year-old Chinese woman complaining of swallowing obstruction, heartburn, regurgitation of gastric acid for more than 2 months. An esophageal lesion was found by chest CT scans in T7 vertebral level. The diagnosis by gastroscopic biopsy was squamous cell carcinoma (SCC) with EGFR over-expression. Simultaneously, chest CT showed a 2 cm x 1 cm solitary lesion in the right superior pulmonary. The histological diagnosis by percutaneous lung Biopsy was “adenocarcinoma.” Epidermal growth factor receptor (EGFR) gene mutation status was evaluated by Sanger sequencing, and an exon 21 point mutation (L858R) was identified. When the double primary malignant tumors were diagnosed, the patient refused operation and received a tyrosine kinase inhibitor (TKI), icotinib, at the dose of 125 mg, three times per day. All serum tumor biomarkers such as CEA and cancer antigen 125 (CA125) were in the normal range during the treatment period. After five-year follow-up, the patient has no evidence of recurrence or metastasis. The lung cancer was stable, meanwhile the esophageal lesion was almost cured.

**Conclusion:**

Icotininb is an effective treatment in the patients of the double primary malignant tumors of lung adenocarcinoma with EGFR gene mutation and esophageal squamous cell carcinoma with EGFR over-expression.

## Background

Lung cancer is still the leading cause of cancer morbidity and mortality in men, but in women, it ranks third for incidence, after breast and colorectal cancer, and second for mortality, after breast cancer. Esophageal cancer ranks seventh in terms of incidence and sixth in mortality all over the world (1). The incidence of esophageal carcinoma associated with lung carcinoma is rare. Hiromichi Ishii, MD reported 0.54% of patients with esophageal carcinoma had associated primary lung carcinoma (2). Surgical treatment for double carcinoma has a high operative risk, especially in older patients. Here, we reported an elderly patient of lung adenocarcinoma with esophageal squamous cell carcinoma with a good outcome by using icotinib.

## Case presentation

### Chief complaints

A 71-year-old Chinese woman was admitted to Bishan Hospital of Chongqing Medical University, complaining of Swallowing obstruction, heartburn, regurgitation of gastric acid for more than 2 months.

### History of present illness

The patient presented with swallowing obstruction, gastroesophageal reflux symptoms including heartburn and regurgitation of gastric acid. She denied any respiratory symptoms such as cough, sputum production, hemoptysis, or fever. Additionally, she had no history of chest or back pain and did not report any other comorbidities.

### History of past illness

The patient had no prior medical history.

### Personal and family history

The patient did not have a cancer-relative family susceptibility.

### Physical examination

Physical examination revealed that the patient’s body temperature was 36.1°C, pulse rate was 70 bpm, blood pressure was 131/82 mmHg, respiratory rate was 18 breaths/min, and performance status score was 1. None of positive signs were found upon physical examinations.

### Laboratory examinations

His blood count showed a WBC of 4.29 × 10^9^/L, Hb of 129 g/L, and platelet count of 188 × 10^9^/L. The serum level of carcinoembryonic antigen (CEA) was 0.98 ng/mL.

### Imaging examinations

**Figure 1:**
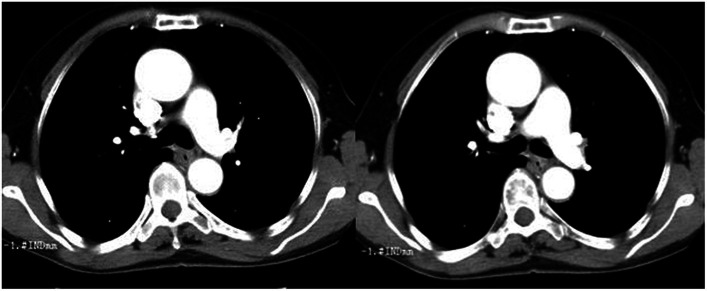
Chest computed tomography (CT) showed an esophageal lesion was found in T7 vertebral level.

**Figure 2:**
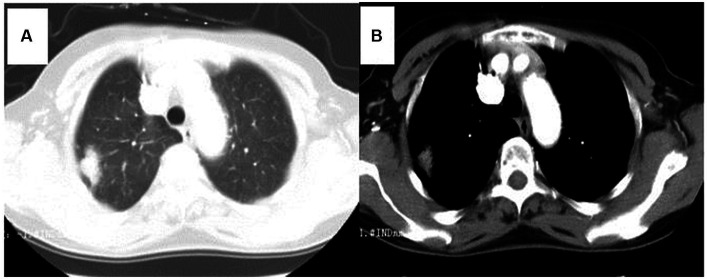
Chest computed tomography (CT) showed a 2 cm x 1 cm solitary lesion in the right superior pulmonary. **(A)** Lung window, **(B)** Mediastinum window.

An esophageal lesion was found by chest computed tomography (CT) scans in T7 vertebral level (Figure 1). Simultaneously, chest CT showed a 2cmx1cm solitary lesion in the right superior pulmonary (Figure 2). The presence of distant metastases was not detected through imaging examinations including abdominal CT scans, brain magnetic resonance imaging (MRI), and bone scanning at the time of diagnosis of the double primary malignant tumors.

### Pathology and genetic testing

**Figure 3:**
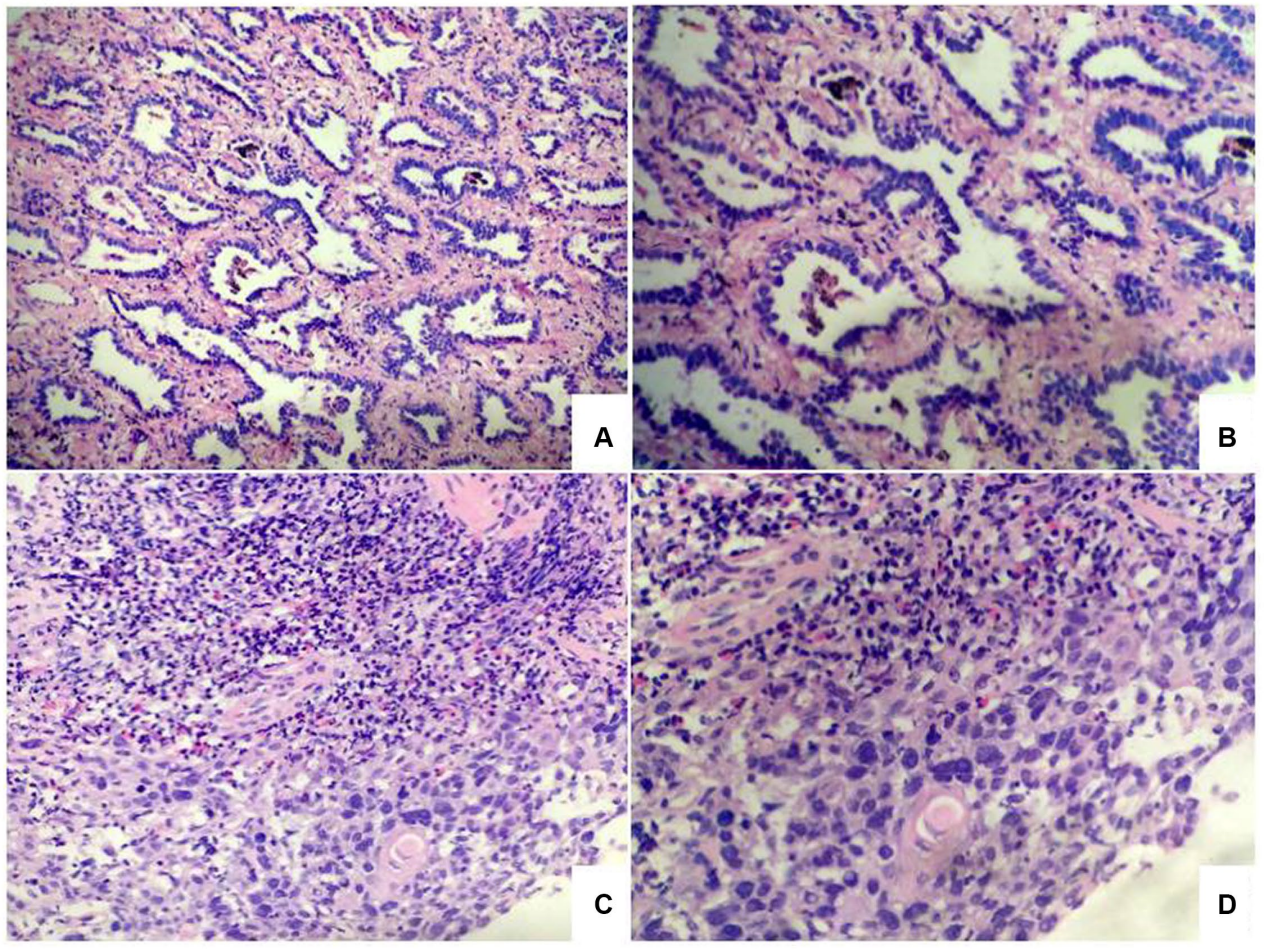
Photomicrographs of lung tumor cells showed adenocarcinoma **(A,B)**. Photomicrographs of esophageal tumor cells showed squamous cell carcinoma **(C,D)**.

The diagnosis by gastroscopic biopsy was squamous cell carcinoma (Figure 3) at the upper thoracic esophagus. Immunohistochemistry indicated EGFR over-expression. Simultaneously, the histological diagnosis by percutaneous lung Biopsy was “adenocarcinoma” (Figure 3). Epidermal growth factor receptor (EGFR) gene mutation status was evaluated by Sanger sequencing, and an exon 21 point mutation (L858R) was identified.

## Final diagnosis

Finally, the patient was diagnosed with stage II squamous cell carcinoma of upper thoracic esophagus (T2-3N0M0) and stage IA adenocarcinoma of the lung (cT1aN0M0 in accordance with version 7 of TNM staging).

## Treatment

When the patient was diagnosed with double primary malignant tumors, she declined surgical intervention, radiotherapy, and chemotherapy. She received a tyrosine kinase inhibitor (TKI), icotinib, at the dose of 125 mg, three times per day. The first-generation TKI, Icotinib, demonstrates a favorable cost advantage compared to other TKIs while exhibiting significant anti-tumor efficacy in NSCLC and ESCC based on previous studies.

## Outcome and follow-up

During the course of treatment, the patient underwent regular blood routine examinations, liver and kidney function tests, and serum tumor marker detections every month. Additionally, she received chest and upper abdominal computed tomography (CT) scans every three months and brain magnetic resonance imaging (MRI) scans every six months. After 2 months of treatment, the patient’s swallowing obstruction disappeared. Between October 2015 and September 2020, all serum tumor biomarkers such as CEA and cancer antigen 125 (CA125) were in the normal range during the treatment period. The lung cancer was stable, meanwhile the esophageal lesion was almost cured. Notably, no adverse effects such as myelosuppression, hepatic or renal dysfunction, diarrhea or rash were observed throughout the treatment. Throughout the course of icotinib treatment, she diligently adhered to medication and regularly attended follow-up appointments.

**Figure 4:**
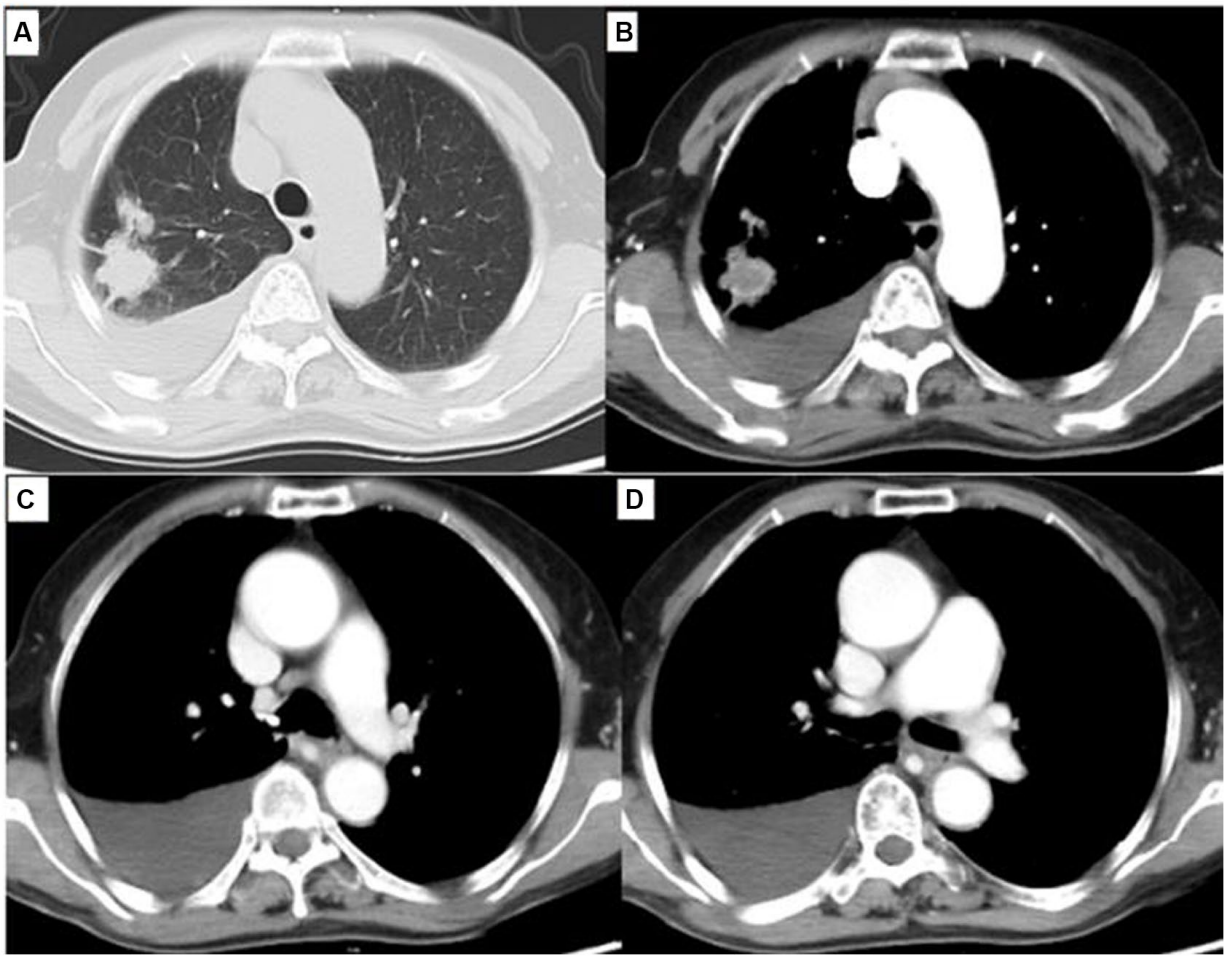
Chest computed tomography (CT) showed a 2.5 cm × 2.1 cm solitary lesion in the right superior pulmonary and moderate pleural effusion. **(A)** Lung window, **(B)** Mediastinum window. Simultaneously, chest CT showed esophageal lesion was almost cured **(C,D)**.

After reimaging in December 2020, CT revealed a 2.5 × 2.1 cm enlarged right superior lobe mass and pleural effusion (Figure 4). The progression free survival (PFS) time was 62 months. When progressive lesions were detected in the lung through CT scan, we recommended the patient undergo a biopsy of the tumor or liquid to identify resistance mutations. However, she declined tissue biopsy and opted for treatment adjustment.

On January 20, 2021, the patient was admitted to the hospital due to shortness of breath after activity. Color Doppler ultrasound showed massive pleural effusion, and the symptoms were improved after pleural puncture and drainage. During hospitalization, the patient felt cervical and thoracic spine pain, and MRI showed C6-C7 cervical and T8-T10 thoracic spine metastasis. Vertebral radiotherapy was suggested, but the patient refused.

Unfortunately, the patient suffered a lower limb fracture due to a fall in April 2021, and she refused surgical treatment. Eventually, the patient died of cachexia in May 2021. The overall survival (OS) time was 67 months.

## Discussion

Lung cancer remains the leading cause of cancer mortality all over the world (1). It is classified into non-small cell lung cancer (NSCLC) and small cell lung cancer (SCLC) clinically. The standard of treatment for patients with stage I and II NSCLC, as well as select patients with stage IIIA NSCLC, is surgical resection. Patients may be offered adjuvant systemic therapy after surgical resection (3). EGFR mutations are detected in up to 50% of lung adenocarcinoma patients (4). Classical activating mutations (exon 19 deletions and the L858R point mutation) comprise the great majority of EGFR mutations, and they are defined as strong predictors for excellent clinical response to EGFR TKI (5). The first-generation TKIs include erlotinib, gefitinib, and icotinib. Afatinib and dacomitinib belong to the second-generation ErbB family blockers. Compared with the first- and second-generation TKIs, third-generation EGFR inhibitors display a significant advantage in terms of patient survival (4).

Esophageal cancer (EC) has a high morbidity and mortality including squamous cell carcinoma (SCC) and adenocarcinoma (6). The dominating type in China is esophageal squamous cell carcinoma (ESCC). Surgery is the primary treatment for EC. A research indicated that surgery was associated with age stratification, and the prognosis was poor in stages I and II patients older than 60 years of age (7).

We report a case of double carcinoma of lung adenocarcinoma and esophageal squamous cell carcinoma. Surgical treatment is the best choice for resectable lung adenocarcinoma or esophageal squamous cell carcinoma, but surgical treatment for double carcinoma has high operative risk, especially in elderly patients. When the double primary malignant tumors were diagnosed, the patient refused operation, radiotherapy and chemotherapy. She received a tyrosine kinase inhibitor (TKI) of icotinib.

Icotinib is the first-generation TKIs, which exerts a good anti-tumor efficacy on non-small cell lung cancer (NSCLC) with exon 19Del and exon 21 L858R mutation (8). It also has a good anti-tumor efficacy in ESCC. Huang et al. revealed that icotinib showed favorable activity in patients with advanced, previously treated ESCC with EGFR over-expression or amplification (9). They included 54 patients with previously treated, confirmed advanced ESCC histologically and EGFR overexpression (immunohistochemical staining sore of 3+) or amplification (positive fluorescence *in situ* hybridization result) in this phase 2, single-arm, multicenter trial. These patients received oral icotinib which was well tolerated overall. Nine responses were observed, including one complete response and eight partial responses, resulting in a 16.7% objective response rate. Sixteen patients had stable disease. The disease control rate was 46.3%. The median PFS time 52 days and OS time was 153 days (9). Luo et al. also confirmed older patients with ESCC benefitted from icotinib. In this randomized, multicenter, open-label, phase II clinical trial, 127 patients aged 70 years or older with clinical stage T2 to T4, N0/1, M0/1a unresectable ESCC were randomized 1: 1 to receive RT plus icotinib or RT alone. Patients treated with icotinib plus radiotherapy had a median OS of 24.0 months, whereas those treated with radiotherapy alone had a median OS of 16.3 months. Furthermore, patients with EGFR overexpression benefitted more from icotinib with RT (10). Wang et al. suggested that EGFR overexpression might potentially be used in predicting the efficacy in ESCC patients treated with Icotinib. In this study, 62 patients received icotinib. The response rate was 17.6% with high EGFR-expressing patients, which was higher than the rate (0%) for patients with low to moderate EGFR-expressing tumors. Moreover, all cases responded to icotinib showed EGFR overexpression (11).

Erlotinib and gefitinib are also the first-generation TKIs, and several studies have confirmed the efficacy on esophageal cancer.

A randomized phase 3 trial revealed patients with overexpressing EGFR treated with erlotinib had a better PFS and OS than the patients without erlotinib. Concurrent chemoradiotherapy with ENI (elective nodal irradiation) and/or erlotinib improved long-term survival in locally advanced ESCC (12). For esophageal squamous cell carcinoma patients who cannot tolerate chemoradiotherapy, concurrent erlotinib and radiotherapy are tolerable and effective (13). A retrospective study suggested radiotherapy plus erlotinib should be a preferable modality compared with CCRT, with similar survival outcomes but better treatment compliance and less toxicities (14). In the treatment of erlotinib combined with radiotherapy, patients with epidermal growth factor receptor amplification and never smokers had the longest OS (22.3 and 16.6 months, respectively) (15).

Gefitinib inhibits EGFR via interruption of the EGFR signaling in the target cells (16). Fumikata et al. demonstrated that gefitinib effectively inhibited the growth of ESCC cell lines *in vitro* and *in vivo* (17). Gefitinib can be safely incorporated into an oxaliplatin-based chemoradiotherapy for esophageal cancer (18). CCRT and gefitinib for locoregionally advanced esophagus cancer of patients produced superior survival (19). Maarten L et al. certified that gefitinib has a certain activity in second-line treatment of advanced esophageal cancer. However, the patient outcome was observably better in female patients and in patients with high EGFR expression or squamous cell carcinoma histology (20). A retrospective analysis also revealed the mutation rate of the EGFR of squamous cell carcinoma patients was significantly higher than that of adenocarcinoma patients. The ORR (44.4%) of the EGFR group was significantly higher than the ORR (0.0%) of the wild type group after EGFR TIK (Gefitinib) treatment (21). A phase III, multicenter, placebo-controlled randomized trial revealed that the use of gefitinib as a second-line treatment in unselected esophageal cancer patients does not improve overall survival (OS), but median progression-free survival (PFS) was slightly longer in the gefitinib group than the placebo group [1.57 months vs. 1·17 months, *p* = 0.020, (22)].

Approximately 40–70% of ESCCs show EGFR overexpressed (17). high expression of EGFR is thought to be associated with cell proliferation, invasion, metastasis, chemoradiotherapy resistance, and poor prognosis (11, 17, 23). In OE21 cell, the EGFR TKIs slightly affected epidermal growth factor receptor dimerization as detected by *in situ* proximity ligation assay. In addition, TKIs inhibited ERK1/2, Akt, STAT3, and RhoA activity. This was accompanied by reduced OE21 cell migration, induction of focal adhesions, and actin cytoskeleton reorganization. Therefore, TKIs limit ESCC cell motility and migration (24).

In our study, the patient received a tyrosine kinase inhibitor (TKI) of icotinib because it was less expensive than gefitinib and erlotinib. Compared with these findings, more encouraging treatment outcome was observed in our case. The patient has no evidence of recurrence or metastasis after 5 years follow up. The lung cancer is stable, meanwhile the esophageal lesion is almost cured. After 62 months of treatment, CT revealed progressive lesions in the lung. The PFS of the double cancer patient with EGFR overexpression and mutation was 62 months, indicating that EGFR-targeted therapy in ESCC is promising and worthy of further exploration. When progressive lesions were detected in the lung through CT scan, we recommended that the patient undergo a biopsy of the tumor or liquid to identify resistance mutations. However, she declined tissue biopsy and opted for treatment adjustment. Eventually, the patient developed vertebral metastases. The limitation of this case was the absence of tumor or liquid biopsy following disease progression. Nevertheless, our inclination was toward considering that the vertebral metastasis originated from the lung cancer due to stability observed in esophageal cancer lesion.

In conclusion, icotininb is an effective treatment in the patients of the double primary malignant tumors of lung adenocarcinoma with EGFR gene mutation and esophageal squamous cell carcinoma with EGFR over-expression. Elderly patients who are not candidates for chemoradiation or surgery may benefit from targeted agents. In the future, further prospective studies are needed to testify our findings.

## Data availability statement

The original contributions presented in the study are included in the article/supplementary material, further inquiries can be directed to the corresponding author.

## Ethics statement

The study was approved by the ethics committee of The People’s Hospital of Bishan District Chongqing. The studies were conducted in accordance with the local legislation and institutional requirements. The participants provided their written informed consent to participate in this study. Written informed consent was obtained from the individual(s) for the publication of any potentially identifiable images or data included in this article.

## Author contributions

MD: Writing – review & editing, Writing – original draft, Visualization, Validation, Supervision, Software, Resources, Project administration, Methodology, Investigation, Funding acquisition, Formal analysis, Data curation, Conceptualization. XL: Writing – review & editing, Writing – original draft, Supervision, Software, Methodology, Investigation, Data curation, Conceptualization. HM: Writing – review & editing, Writing – original draft, Software, Formal analysis, Conceptualization. MW: Writing – original draft, Supervision, Software. LS: Writing – review & editing, Writing – original draft, Project administration, Investigation, Formal analysis, Data curation.
